# Body image and compulsive exercise: are there associations with depression among university students?

**DOI:** 10.1007/s40519-022-01374-x

**Published:** 2022-02-18

**Authors:** Klara Edlund, Fred Johansson, Rebecca Lindroth, Louise Bergman, Tobias Sundberg, Eva Skillgate

**Affiliations:** 1 Musculoskeletal and Sports Injury Epidemiology Center, Department of Health Promotion Sciences, Sophiahemmet University, Sophiahemmet Högskola, Box 5605, 114 86 Stockholm, Sweden; 2 Unit of Intervention and Implementation Research on Worker Health, Institute of Environmental Medicine, Karolinska Institutet, Stockholm, Sweden; 3 Department of Psychology, Uppsala University, Uppsala, Sweden

**Keywords:** Body dissatisfaction, Compulsive exercise, Depression, University students

## Abstract

**Purpose:**

Mental health problems among university students have been reported to be significantly increasing and suggested to be associated with college drop-out. Body dissatisfaction and compulsive exercise are both constructs relevant for mental health problems in general and eating disorders in particular. This study examined associations between body dissatisfaction, compulsive exercise and self-reported symptoms of depression among Swedish university students.

**Methods:**

Participants (*n* = 4262) are students in an ongoing cohort study, and data from the baseline assessment were used. Four linear regression models were built to explore the associations between body dissatisfaction, compulsive weight control exercise and depressive symptoms.

**Results:**

Our findings showed that females reported higher levels of body dissatisfaction than males. Body dissatisfaction and compulsive exercise were associated with self-reported symptoms of depression in this non-clinical population. Results showed that compulsive exercise was negatively associated with reported symptoms of depression, while body dissatisfaction was positively associated with symptoms of depression.

**Conclusion:**

In line with previous research, there was a gender difference in body dissatisfaction where females displayed higher levels of dissatisfaction than males.  Body dissatisfaction was  positively associated with reported symptoms of depression, suggesting support of previous research indicating body dissatisfaction to increase mental health problems. Compulsive exercise was negatively associated with symptoms of depression suggesting a behavior negatively reinforced, supporting both constructs to be of interest for reported symptoms of depression in a non-clinical population of Swedish university students.

**Level of evidence:**

III, cohort study.

**Trial registration:**

http://clinicaltrials.gov/ID: NCT04465435.

## Introduction

Mental health problems among university students have been reported to be significantly increasing, impacting health and wellbeing [25, 30], and suggested to be associated with college drop-out [3]. High levels of psychological distress including anxiety, stress and depression have been well documented among medical students [13] and body dissatisfaction [41] and compulsive exercise [60] may be factors associated with this psychological stress.

Body image has been described as a multidimensional construct that encompasses the internalized view one has of one’s body, including perceptions, thoughts, feelings, and attitudes related to physical aspects of the body, such as weight and shape [58]. Body dissatisfaction (negative attitudes toward the own physical appearance) is one aspect of body image [23]. Most research on body image and body dissatisfaction in the past 20 years has primarily focused on females’ idealization of a thin body [45]. However, during the past decade, male body image issues have drawn more attention [5, 39]. The ideal male body is usually characterized be lean muscularity, leading to concerns generally related to being too fat or not muscular enough [44]. A recent meta-analysis by Barnes et al. [4] demonstrated that an association between male body dissatisfaction and anxiety and depression is likely to exist. Muscle tone along with thinness is also a focus for females resulting in concerns about weight, shape, and muscularity [21, 28, 62]. Body dissatisfaction has been strongly linked to eating disorder symptomatology as well as eating disorder relapse [24]. Further, body dissatisfaction has been shown to be related to extreme behaviors with adverse effects, such as restrained eating, purging, and over-exercising [45], and is considered a risk factor for eating disorders [34, 40, 47, 48], a series of risky health behaviors, such as alcohol and drug use, gambling, self-harm [9], and poor psychological health [11, 17, 40].

Empirical longitudinal studies have confirmed that body dissatisfaction predicts depression among females [6, 16, 17, 22, 38, 42, 50]. Most of these studies were carried out in the early 2000s with participants born in the 1980s. Since body dissatisfaction is increasing over time [54, 57], it is crucial to explore the Millennial generations due to the important role of internet, technology, and social media on lifestyles of more recent generations [8]. The study by Bornioli and colleagues [8] lends support to previous theoretical ideas proposed by Stice and Bearman [6, 49, 50] that young women might suffer adverse psychological health conditions due to their dissatisfaction with their body. Further, it was demonstrated in the Bornioli study [8] that the relationship between body dissatisfaction and depression persisted with control for BMI, indicating that body image is a multi-dimensional construct, not determined exclusively by BMI.

As mentioned above, body dissatisfaction has been identified as a risk factor for disordered eating. Furthermore, has body dissatisfaction also been demonstrated to predict later onset of depressive episodes in adolescence [8]. Body dissatisfaction has been shown to be associated with both depression and anxiety in Swedish adolescents [26], and young female adults [52]. Further, symptoms of depression and other mental health symptoms have been shown to be associated with suicidal ideation among UK university students [1]. Sub-threshold symptoms of eating disorders among college students have also been shown to be highly predictive of suicidality [32]. Further, The Healthy Minds Study [32], the largest mental health survey of college populations in the United States with data from 71 712 students randomly selected from 77 campuses, have reported that weight and shape concerns were associated with higher rates of suicidality, even after controlling for psychiatric comorbidity. Thus, body dissatisfaction is closely linked to disturbed eating and depression, among university students and it has been argued that the pervasive issues of body dissatisfaction are public health concerns [8].

Compulsive exercise is characterized by craving for exercise and training along with weight and shape concerns, resulting in uncontrollable excessive behaviors with harmful consequences. There is a persistent continuation to both alleviate withdrawal symptoms (extreme guilt and/or negative affect when unable to exercise) and to avoid the perceived negative consequences of not exercising [35]. Injuries and impaired social relations are some of the harmful health consequences along with eating disorders pathology and depression [31]. Thus, excessive, and compulsive exercise represents a rigidity and an extreme urge to exercise, even when one does not find joy in it [55], and it has been clearly linked to eating pathology [18]. Research assessing the prevalence of compulsive exercise in non-clinical samples, particularly among college students is lacking, although a recent study has reported compulsive exercise to be relatively common among college students and to be associated with poor mental health symptoms and substance use [20]. Among college students, engaging in compulsive exercise has been reported to be associated with greater risk of a concurrent eating disorder [46], and compulsive exercise is broadly conceptualized as a central component in the development of eating disorder symptoms [36, 37]. As previously presented in the literature, body dissatisfaction and compulsive exercise have been linked to mental health problems (e.g., [52], Stroeber and Otto 2006; [60].

The present study is part of an ongoing large prospective cohort study of mental health problems and musculoskeletal pain in university students in Sweden (see below; SUN; http://clinicaltrials.gov/ID: NCT04465435). Several variables are included in the study, such as life-style factors, different aspects of health, and potential problem behaviors, such as substance use and gambling for money, which are planned for upcoming presentations. Body dissatisfaction and compulsive exercise are two factors studied, with the hypothesis, based on the literature, that they will be linked to self-reported symptoms of depression among students. Assessing body dissatisfaction and compulsive exercise to establish whether there is an association with depression in this non-clinical population is a somewhat novel approach that could be of importance for future causal analysis to better understand how these constructs play a part in depression among students. Thus, the aim of this study was to explore the associations between compulsive exercise, body dissatisfaction and self-reported symptoms of depression in a large sample of Swedish university students.

### Materials and methods

#### Source population, inclusion criteria, recruitment and data collection

The Sustainable UNiversity life study, the SUN-study, is a cohort study of undergraduate- and graduate (up to master level) university students in Sweden. Eligible for participation include students enrolled in full-time educational programs with at least one academic year before planned graduation. This study include 4262 students using baseline data from the SUN-study (http://clinicaltrials.gov/ID: NCT04465435; Edlund et al., *submitted*) [14]. The overall aim of the SUN-study is to advance the knowledge about mental health problems and musculoskeletal pain in university students. The source population are students at universities/colleges in the greater Stockholm area and Örebro, attending selected educational programs in health, nursing, medical, and social sciences as well as technology. The universities selected were located close to the work location of the research since the research staffs’ physical presence was an important recruitment strategy for the study.

Students eligible for the study were mainly invited through e-mail. Email addresses were distributed through the universities. All participants provided online informed consent to participate in the study, after receiving online and/or in person information about the purpose and procedure of the study, and that it has been approved by the Swedish Ethical Review Authority (Reference Number: 2019-03276; 2020-01449). Data were collected online with a baseline questionnaire, followed by four follow-up questionnaires sent out every three months during one academic year. The first baseline questionnaire took about 30 min to complete and the follow-up questionnaires somewhere between 10 and 15 min. As a gesture of appreciation, all students participating in the study were offered a one-month free pass to one of the major health club chains in Sweden, ACTIC, to whom a collaboration for sponsorship has been established. The online platforms used for the data collection were SUNET, hosted online by SUNET Artologik, a secure web-based survey system used in Swedish higher education [53]. The system has previously been used by members of our research group [19].

#### Instruments

##### Depression anxiety scale (DASS-21)

The depression anxiety stress scale is a self-report questionnaire used to assess self-reported symptoms of mental health problems. For the present study, only the scale measuring depression was used (DASS-21; [33]. The original DASS consists of 42 items, addressing three different dimensions of symptoms (depression, anxiety and stress) within the last week. The version most widely used is the adapted and shortened version with 21 items, which is used for the present study. DASS-21 has reported adequate psychometric properties (test–retest and internal consistency, a 0.81–0.96 [2, 33], and has been developed to measure symptoms of depression, anxiety and stress in both clinical and non-clinical populations. The Cronbach’s for the depression-subscale α was 0.91 in this sample.

##### Compulsive exercise test (CET)

The compulsive exercise test is a self-report questionnaire designed to explore the emotional, cognitive, and behavioral characteristics of compulsive exercise (CET; [55]. It comprises 24 items answered on a Likert scale from 0 (never true) to 5 (always true). The CET consists of five subscales: “avoidance and rule-driven behavior”, “weight control exercise”, “mood improvement”, “lack of exercise enjoyment” and “exercise rigidity”. Two subscales are included in the study: “Weight control exercise” (WCE) and “Avoidance of negative affect and rule-driven behavior” (ARDB), since these subscales have adequate validity (factor analysis) and internal consistency (WCE: a = 0.82, avoidance and rule-driven behavior ARDB: a = 0.87,Plateau et al., 2014). A mean score of ≥ 3.47 for the WCE scale and ≥ 2.75 for ARDB have been reported in a Norwegian sample of eating disorders patients [59]. For the present study, Cronbach’s alpha was 0.83 for WCE and 0.90 for ARDB. The questions assess behaviors for weight loss and emotion regulation using training.

##### Body shape questionnaire (BSQ-8C).

The brief version of the Body Shape Questionnaire (BSQ-8C, 8-item version; [61], based on the full 34-item version [10], was used to assess body dissatisfaction. The questions are reflecting the past four weeks regarding body shape concerns with questions addressing e.g., fear of becoming fat, self-consciousness about shape when in company with others and feelings of being excessively large and rounded. A score < 19 indicates no concern with shape, between 19 and 25 mild concern, 26–33 moderate concern and > 33 marked concerns with shape. The eight-item version of BSQ has been found to be sufficiently reliable and valid [15], and the short versions capture the one-factor structure of the full version BSQ [29]. Welch and colleagues (2012) used the BSQ-8C Swedish version as a stand-alone measurement of body dissatisfaction and found it to show high internal consistency of ⍺ = 0.94, excellent test–retest properties. In the present study population, Cronbach’s alpha was 0.93.

#### Statistical analysis

Spearman’s correlations between BSQ and the subscales of CET were calculated. Cohen’s *d* was used as a measure of effect size. Four linear regression models were built to explore the associations between body dissatisfaction, compulsive weight control exercise and depressive symptoms. The first two models explored bivariate associations by regressing depressive symptoms on body dissatisfaction and compulsive weight control exercise, respectively. The third model included both body dissatisfaction and compulsive weight control exercise to explore each of the independent variables' association to depression while holding the other constant. In the fourth model, an interaction-term between compulsive weight control exercise and body dissatisfaction was added to explore whether body dissatisfaction modified the association between compulsive weight control exercise and depressive symptoms. All regression models were adjusted for gender (male vs not male), age (continuous) and level of parental education (at least one parent with university education vs no parent with university education). The BSQ, CET and DASS-21 were all treated as continuous variables in the regression models. Multicollinearity was assessed by the variance inflation factor (VIF). A VIF below ten was regarded as satisfactory.

*Ethical considerations*

Prior to the data collection, the Universities’ Educational Support Offices and Student Health Services were informed about the study and granted approval to implement the study at each site. Before inclusion in the study, informed consent was given by all participants. The study was approved by the Swedish Ethical Review Authority (Reference Number: 2019-03276; 2020-01449).

### Results

A total of 18,973 students were invited to the study and 4263 (23%) participated by completing the web-based survey. See Table 1 for sample demographics.

**Table 1:** Characteristics of full sample and divided by males and females

	Full sample (*n* = 4263)	Males (*n* = 1592)	Females (*n* = 2645)
Age, M (SD)	24.6 (6.1)	23.8 (5.4)	25.1 (6.4)
Gender, *n* (%)			
Female Male Other	2645 (62%) 1592 (37%) 26 (1%)	– –	– –
Year of study			
1st 2nd 3rd Masters	1733 (41%) 936 (22%) 659 (15%) 935 (22%)	614 (39%) 320 (20%) 256 (16%) 402 (25%)	1108 (42%) 608 (23%) 399 (15%) 530 (20%)
Type of education, *n* (%)			
Medical Technical Social sciences	1966 (46%) 1770 (42%) 527 (12%)	458 (29%) 956 (60%) 178 (11%)	1493 (56%) 805 (30%) 347 (13%)
Place of birth, *n* (%)			
Sweden Nordic countries Europe Outside europe	3337 (78%) 138 (3%) 263 (6%) 525 (12%)	1250 (79%) 35 (2%) 97 (6%) 210 (13%)	2068 (78%) 102 (4%) 163 (6%) 312 (12%)

*SD* standard deviation, *n* number

#### Body dissatisfaction

Recalculated scoring for BSQ-8C, based on Taylor [56] is shown in Table 2.

**Table 2:** Percentage dispersion of levels of concern with shape (*n* = 4263)

	Score	Total *n* = 4263 *n*, (%)	Female *n* = 2645 *n*, (%)	Male *n* = 1592 *n*, (%)
No	< 19	2635 (62%)	1337 (51%)	1285 (81%)
Mild	19–25	664 (16%)	488 (18%)	173 (11%)
Moderate	26–33	552 (12%)	426 (16%)	92 (6%)
Marked	> 33	442 (10%)	394 (15%)	42 (3%)

On average, female participants scored higher on the BSQ (mean (M) = 21.3, SD = 10.2, SE = 0.2), than male participants (M = 14.7, SD = 7.1, SE = 0.2). This difference, 6. 7, 95% CI [6.2, 7.2], was significant *t*(4153.5) = 25.1, *p* < 0.0001; with a medium to large effect size, *d* = 0.7. Thus, women reported higher levels of body dissatisfaction than men.

#### Compulsive exercise

On average, females scored higher on Weight Control Exercise (M = 6.6, SD = 4.9, SE = 0.1), than males (M = 5.2, SD = 4.1, SE = 0.1). This difference, 1.4, 95% CI [1.2, 1.7], was significant *t*(3820.5) = 10.2, *p* < 0.0001; although the effect size was small, *d* = 0.3. Thus, a significant gender difference was found, where women more often self-reported to exercise for weight and shape reasons than men. On average, females also scored higher on Avoidance of Negative Affect and Rule-Driven Behavior (M = 9.01, SD = 6.89, SE = 0.13) than males (M = 8.36, SD = 6.63, SE = 0.17). This difference, 0.65 95% CI [0.24; 1.07], was significant *t*(3453.1) = 3.06, *p* = 0.002, with a small effect size *d* = 0.1. Moderate correlations were shown between body dissatisfaction and avoidance of negative affect and rule-driven behavior (*r* = 0.32) and finally, moderate correlations were found between avoidance of negative affect and rule-driven behavior and weight control exercise (*r* = 0.49), and between body dissatisfaction and weight control exercise (*r* = 0.64) (Table 3).

**Table 3:** Correlations between compulsive exercise and body dissatisfaction. Spearman’s rank correlations with 95% CI, (*n* = 4263)

	BD*	WCE**	ARDB***
BD	1		
WCE	0.64 (0.62–0.66)	1	
ARDB	0.32 (0.29–0.35)	0.49 (0.29-0.35)	1

*BD body dissatisfaction

**WCE weight control exercise

***ARDB avoidance and rule-driven behavior

#### Depression

On average, depression levels were higher among females (M = 4.93 SD = 4.73, SE = 0.09) than males (M = 4.42 SD = 4.65, SE = 0.12). This difference, 0.5 95% CI [0.21; 0.79] was significant *t*(3396.7) = 3.37, *p* = 0.0007 with a small effect size *d* = 0.1.

#### Association with reported symptoms of depression

Table 4 shows the association between dissatisfaction (BD), weight control exercise (WCE) and level of depression, respectively. Model 1 showed that body dissatisfaction (BD) was positively associated with higher mean level of depression. Model 2 showed that also compulsive weight control exercise (WCE) was positively associated with mean level of depression. In model 3 (including both BD and WCE) the association between compulsive weight control exercise and depressive symptoms became negative, while the effect of body dissatisfaction on level of depression became more pronounced compared to model 1. Model 4 showed that there was a statistically significant interaction between body dissatisfaction and compulsive weight control exercise, although this interaction is too small to be considered practically significant.

**Table 4:** Linear regression models of the association between compulsive training, body dissatisfaction, and depressive symptoms

	Beta	Std. error	*t* value	*p* value	95% CI
Model 1 (BD*)					
BD	0.18	0.21	6.84	< 0.001	
Model 2 (WCE**)					
WCE	0.16	0.02	10.34	< 0.001	
Model 3 (BD and WCE)					
BD	0.23	0.01	23.77	< 0.001	
WCE	− 0.15	0.02	− 7.82	< 0.001	
Model 4 (BD*WCE)					
BD	0.20	0.01	13.58	< 0.001	
WCE	− 0.26	0.04	− 7.27	< 0.001	
BD*WCE	0.005	0.001	3.59	< 0.001	

*BD  body dissatisfaction (Body Shape Questionnaire)

**WCE weight control exercise

**Fig. 1:**
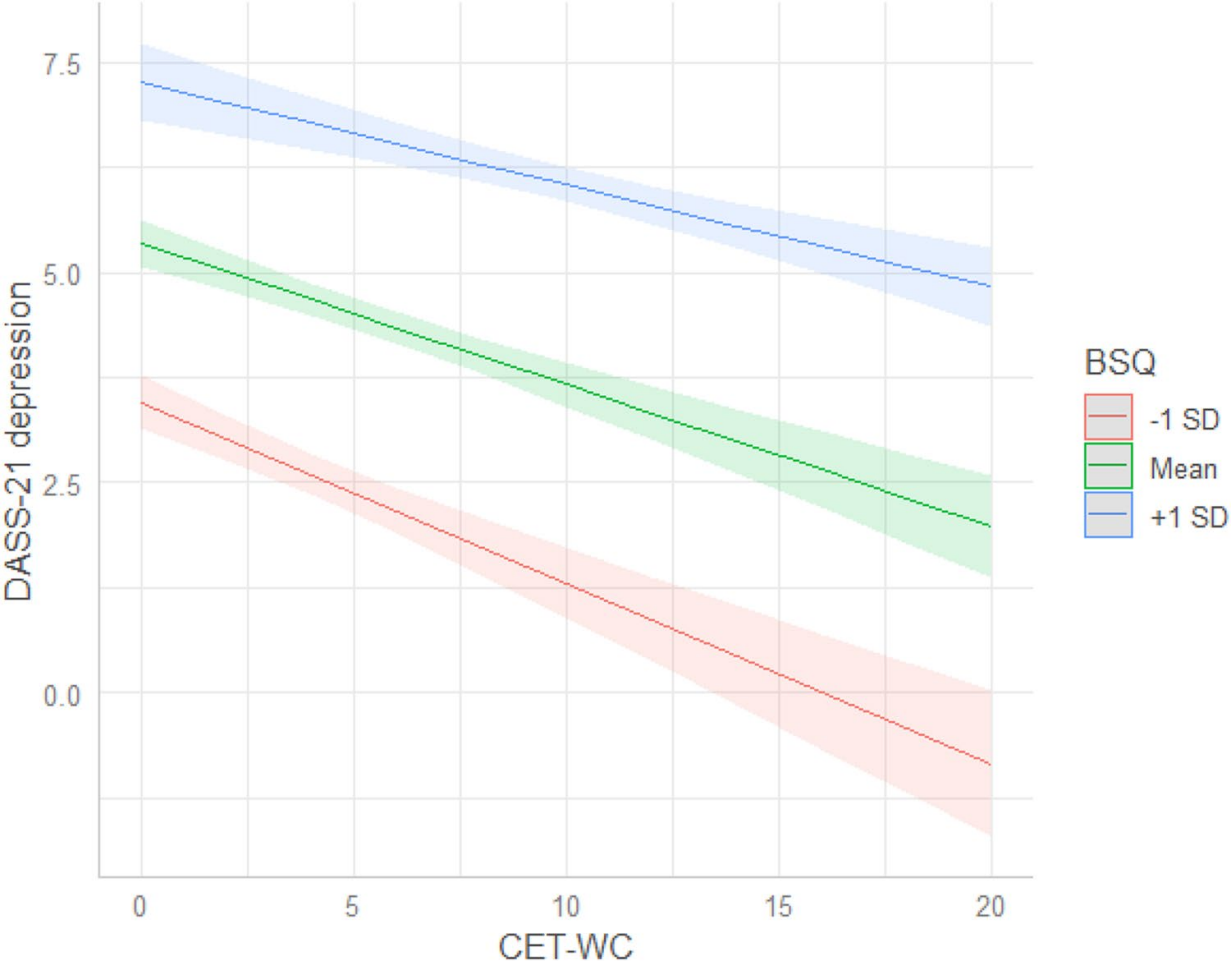
Association between levels of BD, WCE and level of depression. The figure shows predicted values of depressive symptoms from model 4.The lines represent different levels of BD, see legend

Both BD and WCE were associated with depressive symptoms. When adding both predictors in the same model, WCE was negatively associated to depression symptoms, while BD was still positively associated. This indicates that within levels of BD, WCE is negatively associated to depression (Fig. 1).

### Discussion

The aim of this study was to explore the associations between compulsive exercise, body dissatisfaction and self-reported symptoms of depression among Swedish university students.

#### Results

Our results confirmed previous research showing female students to display more body dissatisfaction than male students, with 31% of the females and 9% of the males reported moderate or marked levels of body dissatisfaction. Both body dissatisfaction and compulsive exercise were positively associated with self-reported symptoms of depression, when modeled separately. However, when modeled jointly, compulsive exercise showed a negative association with self-reported symptoms of depression, whereas body dissatisfaction showed a positive association. Regarding exercise for weight control and self-reported symptoms of depression, higher levels of weight control were related to lower symptoms of depression when adjusting for body dissatisfaction. Correlational analysis showed that all variables used in the multiple linear regression model were independent constructs. Since the correlations were found to be significant, even though the correlation not perfect, we argue that the constructs were reasonable to include in the model. Our results suggest body dissatisfaction and compulsive exercise to be associated with self-reported symptoms of depression among a non-clinical sample of university students.

Our results are in line with previous reports of excessive exercise being associated with different psychological traits where poor emotion regulation and compulsivity are two of them [12]. Previous studies have suggested compulsive exercise to be a dysfunctional emotion regulation strategy [43]. We suggest that these behaviors in the short run may serve as dysfunctional strategies, maintained by processes of negative reinforcement, where individuals continue to exercise as a result of the removal of aversive stimuli, such as negative emotions [7]. These results indicate that these variables are constructs of importance in a non-clinical population regarding self-reported symptoms of depression.

#### Strengths and limitations

A strength of the study is that it is based on a large sample from the baseline measure of a cohort study with more than 4000 students included. The use of well-established instruments and an easily accessible digital platform for our web-based surveys are also strengths. A limitation of the study may be that all participating students were enrolled at universities in the Stockholm area which may limit the generalizability of the results. Further, only 23% of the invited students participated by completing the web-based survey. This is a potential threat to the validity of the study, but since the aim was to study associations, and not to primary report on the occurrence, we believe this potential bias is not extensive.

#### Clinical relevance

Body dissatisfaction and compulsive training are core features of eating disorders, and body dissatisfaction has also been considered to increase the risk of developing eating disorders [34, 40, 47, 48]. The co-morbidities with eating disorders and other mental health problems, such as depression, are high. By studying associations between body dissatisfaction, compulsive training, and symptoms of depression, we have identified a positive relation between body dissatisfaction and symptoms of depression along with a dysfunctional strategy for emotion regulation, compulsive training. Our results suggest both constructs to be of interest for reported symptoms of depression in a non-clinical population of Swedish university students, and are, to our knowledge, novel results.

#### Future research and implications

To fully understand the relationship between body dissatisfaction and physical activity, further research is needed to understand the potential relationship between not only body dissatisfaction but also positive body image and physical activity [27]. Output from such research may help differentiate between functional and dysfunctional behaviors and attitudes related to body image and exercise, which could be of fundamental importance to the understanding of risks for developing psychopathologies.

The cross-sectional design used in the present study does not lead to understanding the direction of the associations between the study variables. Therefore, longitudinal analyses are needed to understand causal associations between compulsive exercise, body dissatisfaction and depression among students. Thus, data included in the present study are from the baseline measure of the ongoing cohort study SUN, lending the opportunity for further analyses to study changes in these variables prospectively over a one-year-period of time.

What is already known on this subject? That body dissatisfaction is a core clinical feature in eating disorders and a well-established risk factor for the development of pathological eating patterns and disorders. What does the study add? The indications of the association between body dissatisfaction, compulsive training, and depression in a non-clinical population of university students. Further that compulsive training was negatively associated with depression suggesting compulsive training to serve as a dysfunctional strategy for emotion regulation.

## Conclusion

In line with previous research, there was a gender difference in body dissatisfaction where females displayed higher levels of dissatisfaction than males. Body dissatisfaction was positively associated with symptoms of depression, suggesting support of previous research indicating body dissatisfaction to increase mental health problems. Compulsive exercise was negatively associated with symptoms of depression suggesting a behavior negatively reinforced, supporting both constructs to be of interest for reported symptoms of depression in a non-clinical population of Swedish university students.

## Data Availability

The dataset generated and analyzed during the current study are not publicly available due to secondary confidentiality and privacy of the participants.
